# Legal and Ethical Issues in Periviable Decision-Making in the Current Moment

**DOI:** 10.1017/jme.2025.10186

**Published:** 2025

**Authors:** Elizabeth Tobin-Tyler, Brownsyne Tucker Edmonds, Erika Rose Cheng

**Affiliations:** 1 Health Services, Policy and Practice, Brown University School of Public Health, United States; 2 Department of Pediatrics, Indiana University School of Medicine, United States; 3 Department of OB/GYN, Indiana University School of Medicine, United States

**Keywords:** Periviable delivery, Decision-making, Post-Dobbs, Diverse families, Medical ethics, parental rights laws, resuscitation

## Abstract

Periviable births, occurring between 20 and 25 weeks of gestation, present significant challenges due to varying survival rates and potential morbidities for survivors. Medical decision-making in this context raises ethical and legal questions, including considerations of sanctity of life versus quality of life and challenges in the clinician-parent relationship. This article outlines the complex ethical and legal landscape surrounding parental medical decision-making for periviable infants in the United States, discussing the evolution of federal and state laws. Existing laws highlight a vitalist approach that prioritizes life preservation despite potential harm and overlook non-heteronormative and non-traditional family structures, complicating decision-making. The impact of post-Dobbs state abortion bans on parental and clinician autonomy have exacerbated these challenges. We advocate for legislative support for inclusive definitions of legal parenthood to facilitate evidence-based decision-making centered on patients and families. Also needed are legal frameworks that accommodate the intricacies of periviable birth decisions while respecting patient autonomy and medical expertise, especially amidst the evolving legislative environment.

## Introduction

The American College of Obstetricians and Gynecologists reports that approximately 0.5% of births in the United States occur before the third trimester of pregnancy.^1^ Periviable births are those that occur between 20 0/7 and 25 6/7 weeks gestation and for which infants are “at the threshold of viability.”^2^ Studies of periviable births demonstrate significant variation in survival rates — from 0% to 37% at 22 weeks to 59% to 86% at 25 weeks of gestation.^3^ Extremely premature infants, if they survive, are likely to experience significant morbidities and neurodevelopmental impairment.^4^ When an infant must be delivered very prematurely, parents, in consultation with their health care teams, are faced with medical decisions that can be both ethically challenging and emotionally taxing. One such decision prior to birth is whether or not to resuscitate a periviable infant at birth.

Medical decision-making in the context of periviable birth raises a range of both ethical and legal questions. In situations in which survival is unclear, ethical considerations include weighing sanctity of life against quality of life. Vitalism — preservation of life at all costs — does not fully consider the potential harms of intervention to the newborn.^5^ A best interests approach, on the other hand, attempts to consider the relative benefits and burdens of intervention for the infant, including likelihood of short and/or long-term pain and suffering and severe impairment.

Ethical issues also arise regarding the clinician-parent relationship. Biomedical ethics dictate that clinicians respect patients’ autonomous medical decisions. In the case of parental medical decision-making for a periviable infant, clinical counseling in the face of an uncertain prognosis, often in exigent circumstances, can challenge the clinician-parent relationship. Shared decision-making, which “involves clinicians and patients together evaluating the evidence of intervention risks and benefits; considering patients’ preferences, goals, values, and concerns; and arriving at a decision” is considered best practice.^6^ But in the case of decision-making for the treatment of a periviable infant, shared decision-making should include not only the pregnant person but also their partner, as both bear the repercussions of the decision.

Laws in the United States governing treatment of periviable or otherwise medically fragile newborns have been largely shaped by the concept of “abortion exceptionalism,”^7^ — that is, legal deviations from generally accepted principles when fetal life is involved. Although decision-making during pregnancy about resuscitation of a periviable infant does not implicate pregnancy termination, laws in the United States governing treatment of periviable or otherwise medically fragile newborns have been influenced by a vitalist approach that seeks to preserve fetal, and by extension, newborn life at the expense of respect for medical expertise and patient or parental decision-making authority. However, in practice, the law continues to be in tension with the realities of clinical and parental decision-making around periviable birth.

Furthermore, as family structures have become more diverse, the law has lagged — sometimes intentionally and sometimes unintentionally — behind the reality of parenting relationships that do not fit a traditional heteronormative model. This failure to recognize non-traditional families — such as non-biologically related LGBTQ parents, adoptive parents or those engaging surrogates — complicate clinical engagement of parents who may have a stake in decision-making.

Because resuscitation decision-making at periviable gestational ages occurs antenatally (prior to birth), it invokes different ethical and legal questions than postnatal medical decision-making for critically ill neonates or infants. Postnatally, physicians drive the decision-making. They determine if and when interventions are initiated and/or withdrawal of care is allowable based on their assessment of patient prognosis and the treatment’s medical benefit or futility. Antenatally, where overall prognosis is poor, but outcomes are both variable and difficult to predict, parents, not physicians, make the decision about whether to initiate intensive care or palliative care based on their values and goals of care. In addition to the added prognostic uncertainty of the antenatal period, greater uncertainty also surrounds parental status and decisional standing in the antenatal setting, before birth certificates are signed or parental rights are terminated. These complexities raise questions such as: How should the clinician counsel parents in the face of medical/prognostic uncertainty? How should state laws governing parentage and fetal status be applied in these cases? Who (which parents) should be engaged in decision-making?

This article considers the ethical and legal questions that arise in the context of parental medical decision-making for periviable infants. First, it tracks the development of federal laws supporting and limiting parental and clinical decision-making, especially in the context of treatment of periviable infants. Second, it describes state law definitions of legal parenthood, state parental consent to treat laws, and the potential implications of post-*Dobbs* state abortion restrictions, such as whether states may move to restrict parents’ and clinicians’ ability to make treatment decisions for periviable newborns. Third, it highlights ethical and practical challenges inherent in shared decision-making between clinicians and parents involving resuscitation of periviable infants. Finally, it considers how non-heteronormative and non-traditional parents may or may not be engaged in these decisions, as well as the legal and ethical challenges that may arise when parents disagree. It concludes by discussing future considerations for managing complex issues related to clinical and parental decision-making about treatment of periviable infants in the current political climate.

## Federal Law

### Constitutional Protections for Parental Decision-Making

The rights of parents to make medical decisions for their children are grounded in decisions by the Supreme Court, including *Meyer v. Nebraska* ^8^ and *Pierce v. Society of Sisters*,^9^ when the Court found that parents have a fundamental liberty interest under the Due Process Clause of the Fourteenth Amendment to control the upbringing of their children and to direct their education. The Supreme Court reaffirmed parental rights to make decisions regarding the “care, custody and control” of their children in *Troxel v. Granville* in 2000.^10^

Federal courts have further interpreted parental rights to extend to medical decision-making.^11^ While noting that the parental right to make medical decisions for children is not absolute, the Supreme Court declared in *Parham v. J.R.* that there is a presumption that parents act in their children’s best interests unless there is compelling evidence to the contrary.^12^ Notably, this presumption was recently challenged in the context of gender-affirming care. In the 2025 ruling in *U.S. v. Skrmetti*, the Supreme Court overturned a District Court decision that had held that Tennessee’s gender affirming care ban both violated the Equal Protection Clause by discriminating based on sex and infringed on parents’ fundamental right to make medical decisions for their children. Instead, the Supreme Court sanctioned Tennessee’s power to ban gender affirming care based on its stated objective of protecting minors’ health and welfare, even in cases in which the minor, the minor’s parents and their health care provider believe this care is in the minor’s best interests.^13^ While the constitutional question before the Supreme Court in *Skrmetti* was focused on whether or not the ban violated the Equal Protection Clause based on sex, and not explicitly about parental authority to make medical decisions under the Due Process Clause, the ruling has important implications for limiting parental rights in medical decision-making for their children.

### Withholding Treatments for Premature and Profoundly Disabled Newborns

Although the Supreme Court has mostly affirmed the rights of parents to make decisions about their children’s medical care, the issue of clinical and parental discretion to withhold treatments from profoundly disabled infants or infants born at the limits of viability has generated significant controversy and response from federal lawmakers. Beginning in the 1970s, with the development of medical interventions that could keep premature infants alive, novel legal questions arose: When should parents, in consultation with their clinicians, be able to decide not to resuscitate an infant with a poor prognosis? What is the government’s role in ensuring that disabled infants are not discriminated against when these decisions are made?

Until the early 1980s it was generally assumed that parents should make medical decisions for periviable infants, including whether to pursue resuscitation.^14^ But a controversial Indiana state court decision in 1982, *In re Infant Doe*,^15^ upholding the decision of parents, in consultation with their physician, to refuse corrective surgery for their severely disabled infant with Down syndrome brought the question into public discourse. The infant had a congenital abnormality that prevented food from passing from the esophagus to the stomach; the parents also chose to withhold feeding, to prevent food from entering the infant’s lungs. The case prompted a national debate about parental authority in medical decision-making involving disabled, and later, periviable newborns. In response to the “Infant Doe” case in Indiana and others like it, the Reagan Administration issued the so-called “Baby Doe rules” under Section 504 of the Rehabilitation Act of 1973. The regulations established a hotline through which “[a]ny person having knowledge that a handicapped infant is being discriminatorily denied food or customary medical care should immediately contact” the US Department of Health and Human Services (DHHS).^16^ Hospitals were required to post notices about the toll-free hotline in delivery rooms, maternity wards, nurseries and other units providing care to newborns. The rules also required child protection agencies to establish protocols for responding to allegations of medical neglect of newborns.^17^

However, the Baby Doe rules were short-lived. In 1986 in *Bowen v. American Hospital Association*, the Supreme Court struck down the regulations, saying that there was “nothing in the administrative record documenting the Secretary’s belief that there exists ‘discriminatory withholding of medical care’ in violation of Section 504.”^18^ The Court also held that the law did not give DHHS the authority to “give unsolicited advice either to parents, to hospitals, or to state officials who are faced with difficult treatment decisions.”^19^

Before the Court struck down the Baby Doe rules, Congress had acted on the issue. In 1984, it enacted a new law amending the Child Abuse Prevention and Treatment Act (CAPTA) regulating the withholding of treatment for infants with life-threatening conditions. The law directed medical providers to provide maximal treatment to preserve life, except if: the infant is irreversibly comatose; treatment would simply prolong death; or treatment is “virtually futile” and is inhumane. CPS agencies were charged with investigating violations of law.^20^ DHHS issued final rules implementing the law in 1985.^21^ As legal scholar Craig Conway has noted, critics found these rules “too distant from the reality of clinical decision-making, inconsistent with regulations affecting incompetent adults, or too dismissive of parental autonomy in making medical decisions on behalf of a child.”^22^ Still, others commended the rules as demonstrating the importance of protecting life for people with disabilities.^23^

While the CAPTA rules sought to guide clinical decision-making, they appear to have only created confusion and frustration among neonatologists, who recognized the high probability of death or injury following periviable neonatal resuscitation attempts, such that the treatment could, in many cases, be characterized as prolonging death, “virtually futile,” or inhumane.^24^ In light of the confusion, the American Academy of Pediatrics (AAP) supported a “best interests” of the infant standard for determining when treatment could be withheld. This approach allows parents and clinicians to withhold treatment if they determine it is not in the best interests of the infant, including if the “treatment’s likelihood of advancing those interests are unclear.”^25^

### Parental Decision-Making About Infant Resuscitation Prior to Birth

As national debate continued over the federal government’s role in regulating decisions to withhold treatment from premature or severely ill newborns, the issue of prenatal decision-making arose in the landmark case *Miller v. HCA* in 2003.^26^ In Houston, Texas, a woman went into preterm labor at 23 weeks gestation. After being counseled by her obstetrician and a neonatologist that the fetus’ extremely poor prognosis — including the likelihood of death or severe impairment such as lung disease, brain hemorrhage, blindness and intellectual disability — she and her husband decided that they did not want resuscitation measures taken after birth.^27^ However, after a nurse shared this decision with other medical staff, hospital administrators determined that the neonatologist should assess the newborn after delivery and make a decision about resuscitation at that time. The Millers refused to sign a consent for resuscitation. A few days after birth, the newborn, named Sidney, experienced a brain hemorrhage resulting in severe, permanent impairment. The Millers sued the hospital for battery and negligence, and a jury awarded them damages for medical expenses and punitive harm.^28^

The Texas Court of Appeals overturned the jury’s verdict, prompting the Millers to appeal to the Texas Supreme Court. The central question before the court was whether a clinician has the authority to make a medical judgment in a life-or-death emergency, even when parents have previously expressed treatment preferences. The court sided with the hospital, concluding that prenatal decisions about resuscitation were inherently speculative. It accepted the argument that the neonatologist had to make a “split second decision” at birth based on direct observation of the newborn.^29^ With regard to prenatal decision-making, the court said: “Any decision the Millers made before Sidney’s birth concerning her treatment at or after her birth would necessarily be based on speculation.”^30^ Nevertheless, the court affirmed that clinicians are legally obligated to follow parental medical decisions after birth unless a court order explicitly overrides those decisions.

While *Miller v. HCA* was still moving through Texas courts, Congress passed and President George W. Bush signed the Born-Alive Infants Protection Act (BAIPA) in 2002. BAIPA established that any infant born alive — regardless of gestational age or prognosis — is considered a legal person entitled to full protection under federal law. Notably, the law rejected the “best interests of the infant” standard endorsed by the AAP and medical ethicists, instead framing protection as independent of parental preferences or clinical judgment.^31^ Because BAIPA was passed in the context of debates about “partial-birth abortion” bans, clinicians generally viewed the law as primarily focused on regulating abortion as opposed to neonatal care. The AAP’s Neonatal Resuscitation Program (NRP) issued an opinion clarifying that BAIPA was unlikely to change standard neonatal practice.^32^

In 2005, DHHS announced that it would enforce BAIPA through the Emergency Medical Treatment and Labor Act (EMTALA), which requires emergency departments to provide medical screening and stabilizing treatment for patients with emergency medical conditions.^33^ DHHS interpreted EMTALA to require medical intervention to protect all infants “born alive.”^34^ Like the Baby Doe rules, enforcement of the BAIPA rules relies on medical staff to report suspected violations to DHHS.

As with many controversial laws, a presidential administration’s priorities and philosophy govern enforcement. While enforcement of BAIPA was not a priority for the Obama administration, the Trump administration issued an “Executive Order on Protecting Vulnerable Newborn and Infant Children” in 2020 reiterating its commitment to using EMTALA to enforce BAIPA and other federal laws, including Section 504 of the Rehabilitation Act.^35^ Rather than targeting parental decision-making as BAIPA had by framing the problem as clinicians withdrawing treatment to periviable newborns based on parental preferences for palliation, Trump’s Executive Order paints a picture of parents and clinicians battling over resuscitation decisions wherein physicians refuse to care for the infant “even when parents plead for such treatment.” In response, clinicians argued that the executive order threatened the important evolution in clinical practice since the Baby Doe rules toward shared decision-making between parents and clinicians based on the best interests of the child.^36^

## State Laws Regulating Parental Decision-Making in Medical Care

A wide range of state laws impact parental decision-making in medical care. They govern who is considered a legal parent, who has the authority to make medical decisions for a minor, and the balance between parental rights and state authority to protect a child from medical neglect. These laws seek to balance the interests of parents to control their children’s upbringing, the state’s interests in protecting children’s health and well-being, and a minor’s independent interests in bodily autonomy. State laws, therefore, regulate when a state child protection agency may intervene to protect a child from medical neglect^37^ and when a minor is deemed competent to make medical decisions without parental consent.^38^ In the context of medical decision-making for treatment of a periviable infant, it is important to first understand how state laws have traditionally approached parental medical decision-making and how these laws fail to account for the rights of non-heteronormative parents.

### Who is Considered a Parent With Legal Authority to Make Medical Care Decisions for a Child?

As the types of non-traditional families — in which parents do not conform to a traditional model of heterosexual cisgender married couples with biological children — have expanded over the past few decades, state lawmakers and courts have confronted how to draft or interpret parentage laws as applied to diverse types of family structures. Specifically, they have grappled with three core questions regarding who is a legal parent for the purposes of medical decision-making for children: What role should *biological connection* play in parental rights to make decisions about a child’s or fetus’s medical care? What role should *intention* — the expectation that a non-biological person is or will become the parent through adoption or surrogacy — play in determining who may make medical decisions regarding a fetus or child? What role should *involvement* — or the lack thereof — play in determining parental rights in decision-making? Laws and court decisions have varied across states in determining who is considered a legal parent for purposes of decision-making authority.

Some states’ statutory definitions of “parents” are quite broad, such as New Jersey’s, which includes biological, adoptive and stepparents as well as a legal parent’s “paramour,” or any person who has assumed responsibility for the child.^39^ On the opposite end of the spectrum, Wisconsin defines “parent” as the: (a) biological parent, (b) husband who has consented to artificial insemination of his wife, or (c) a parent by adoption;^40^ Arkansas defines a parent as the: (a) biological mother, (b) adoptive parent, or (c) “a man to whom the biological mother was married to at the time of conception or birth.”^41^ States with these narrower definitions exclude LGBTQ parents from decision-making, who, at the time of birth, have not yet established parental rights through adoption, or partners who are not married to the pregnant person.

The Uniform Parentage Act (UPA), last revised in 2017, parts or all of which may be adopted by states, “provides the prevailing view of how to establish legal parentage in each state.”^42^ It allows non-biological parents in same-sex couples to gain legal status through a voluntary acknowledgment of parentage (VAP),^43^ although parental rights generally vest only after birth.^44^ In surrogacy cases, courts may recognize intended parents during pregnancy if a legal agreement exists, but those rights are enforceable only after birth. Genetic surrogates, in which the surrogate is genetically related to the fetus, have a 72-hour right to rescission after birth.^45^ This provision follows state laws that protect birth parents who plan to relinquish parental rights after birth to facilitate adoption. The UPA also provides recognition for “de facto” parents — those who have acted as parents over time — though this status is similarly unavailable during pregnancy.^46^ Some states that have not adopted the UPA have extended legal recognition to de facto parents through other state laws.^47^ Since “de facto parents” laws are based on relationships established over time between adults and children, they play no role in decision-making during pregnancy.

While the 2017 UPA made great strides in expanding the legal definition of “parent,” as of 2025, it has been enacted in only ten states and introduced in four state legislatures.^48^ Additionally, it does little to address questions related to parental decision-making authority during pregnancy, as generally parental rights are not established until birth.

### State Parental Consent to Treat Laws

Consent to treat laws define who has legal authority to make medical decisions for a child. State statutes usually create a priority list for who may consent. For example, in South Carolina, the order of priority for consenting individuals is as follows: (a) legal guardian, (b) parent, (c) grandparent or adult sibling, (d) other blood relative who the health care professional believes is close with the patient, and (e) other individual — not blood related — who the health care professional believes has a close relationship with the patient.^49^ A non-traditional parent in South Carolina, therefore, would be at the end of the priority list unless they had been adjudicated as a legal guardian. Again, since this adjudication would not occur until after birth, non-traditional parents could be excluded from decisions related to treatment at birth.

However, many states allow a legal parent to authorize another adult to consent to treatment for a minor. This could provide the opportunity for a pregnant person to authorize decision-making to a partner who may not otherwise be recognized under the law.^50^ In emergency situations involving periviable birth, however, preauthorization may not be in place and the non-pregnant parent may not be informed about options.

Furthermore, in the case of medical decision-making for a minor, states generally only require that one parent consent if both parents are not present or one is unavailable. During pregnancy or at the time of birth, the patient is the pregnant person and a clinician may focus their attention on informing the patient of postnatal options for treatment of a periviable infant without engaging the other parent. Because postnatal decisions about treatment of a periviable infant most often occur at the intersection of obstetrics and neonatology, engagement of both parents and the full clinical team best facilitates shared decision-making. In situations in which a partner’s legal status as parent is unclear or not established, some clinicians may hesitate to engage that parent in decision-making. Additionally, research on informed decision-making for prenatal screening has indicated that providers tend to include fathers in discussions only if they happen to be present, despite the preference of most parents for joint decision-making.^51^

Even when both parents are fully informed of the options and engaged in the decision-making process, there is always a possibility that they may disagree about the treatment plan. State laws are virtually silent on this issue. Generally, if parents disagree about a medical decision for a child, court involvement is required to resolve the dispute. When parents disagree about a medical decision that involves a matter of vital importance like a decision to withhold treatment, courts have typically sided with the parent who refuses to consent to the DNR.^52^

### Laws Governing Decision-Making During Pregnancy

State laws provide little guidance regarding parental decision-making during pregnancy for treatment of periviable infants. While abortion statutes govern when a pregnant person may terminate a pregnancy,^53^ they do not address prenatal decisions about postnatal treatment. In the post-*Dobbs* era, however, some states are taking a more aggressive stance, extending legislative reach into broader aspects of perinatal care.

Several state legislatures have introduced bills echoing earlier federal efforts, like BAIPA, mandating treatment for any infant “born alive,” regardless of gestational age or prognosis. These proposals are often fueled by the myth of “post-birth abortions,” a narrative promoted by the anti-abortion movement despite existing homicide laws that already protect newborns.^54^ In 2023, for example, the Arizona legislature passed a bill requiring all “born alive” infants to receive medical treatment, with felony penalties for noncompliant clinicians. Although vetoed by Governor Katie Hobbs,^55^ the legislation signals a broader shift toward politicized oversight of neonatal care. Because parents of periviable infants are faced with decisions about post-birth treatment, including resuscitation versus palliation, laws mandating intervention and limiting prenatal decision-making, regardless of prognosis, can lead to undue suffering. Further, these laws risk undermining shared decision-making, as clinicians may become hesitant to discuss or offer palliative options out of fear of legal repercussions.

## Ethical and Practical Challenges of Shared Decision-Making

Treatment decisions for periviable infants raise thorny ethical questions. Biomedical ethics are governed by principlism grounded in beneficence (acting for the benefit of the patient), nonmaleficence (doing no harm), autonomy (informed patient decision-making that is not coerced) and justice (fair and equitable treatment).^56^ Because these principles may conflict, they must be weighed and applied in the context of the problem presented. In the context of periviable treatment decisions, challenging questions arise about patient autonomy when decisions must be made about postnatal resuscitation. Should the decision be left to the pregnant person as the patient? Clinicians and parents must weigh whether intervention may cause harm — unnecessary suffering for an extremely fragile neonate. Assessing what course of action is in the best interests of a neonate implicates questions about quality of life, sometimes in the face of medical uncertainty. Finally, justice demands that treatment decisions be made equitably and without conflict of interest or bias. Below we explore some of the ethical and practical issues that arise in decision-making for the treatment of periviable infants.

### Best Interests Standard

Although lawmakers have largely taken a vitalist approach — emphasizing life preservation at all costs — the clinical community generally endorses a “best interests of the infant” standard, which in essence requires an assessment of how different options implicate beneficence and nonmaleficence. Practically, the approach weights the likely benefits and burdens of intervention, considering survivability, pain and suffering, and projected quality of life from the child’s perspective, not the parents’.^57^ The AAP supports a shared decision-making framework, in which clinicians provide evidence-based prognostic information and may seek court intervention if parental decisions are deemed inconsistent with the infant’s best interests.^58^

The best interests standard invites flexibility and allows both clinicians and parents to evaluate whether treatment is proportionate to the infant’s needs and likely outcomes — something that is not afforded by a purely vitalist approach. However, it also begs the question: whose definition of best interests should be given greater weight — the clinician or the parents? In situations where prognosis is poor or uncertain, parents, serving as surrogate decision-makers for their infants, often apply their own values and beliefs in determining what is best for their child. As such, the clinician’s role includes not only informing parents about likely prognosis and treatment options, including withholding treatment, but also engaging them in values clarification.^59^

### Ethical Issues Related to Physician-Parent Communication

Unfortunately, studies show that communication between clinicians and parents in decisions about resuscitation of periviable infants is not always optimal. Discussion about palliation, or comfort care, for neonates are understandably fraught and many clinicians remain reluctant to introduce the topic to parents. Unlike decision-making about end-of-life for older adults, parents may feel like it is wrong to give up before a child’s life even begins.^60^ With technological improvements that have allowed for survival at earlier and earlier gestational ages, prognostic uncertainty remains high, and parents remain hopeful. Even in the most dire of cases, parents may hold fast to the “therapeutic illusion” — the belief that, despite a prognosis of death or profound disability, their child will “beat the odds.”^61^ Although there is some evidence that clinicians may avoid conversations about resuscitation,^62^ recent studies show that parents who have had to make treatment decisions for periviable infants report that they felt well-informed by their clinicians about treatment options.^63^

The nature of physician-parent communication, however, is subject to societal forces that invoke questions about equity and justice, particularly in the context of the Black maternal morbidity and mortality crisis in the US. Trust between clinicians and racially marginalized patients has been undermined by the legacy of racism in obstetric care and by persistent reports of racial discrimination and mistreatment.^64^ In 2023, the CDC reported that: “Approximately 40% of Black, Hispanic, and multiracial mothers reported discrimination during maternity care, and 45% of all mothers reported holding back from asking questions or discussing concerns with their provider.”^65^ Another survey of mothers found that Black women report more often than other women that their clinicians do not listen to them.^66^

Furthermore, Black women have the highest rates of pre-term birth,^67^ making it more likely that they will be faced with challenging treatment decisions for a periviable infant. Because Black parents are more likely to be reported to and investigated by Child Protective Service than white parents,^68^ they may also fear that their medical decisions will be reported to CPS by the clinical team, making them less willing to engage in open, honest, and shared decision-making with their clinicians.^69^ Lastly, clinicians may also counsel and share information differentially based on implicit biases that shape assumptions about which patients can understand the complexity of the clinical situation and navigate complex decision-making; assumptions about which neonates’ lives are more valuable or valued (by their families or in general); or assumptions about which families are equipped to care for a child with significant neurodevelopmental impairment. For example, in a study of obstetricians’ counseling practices for periviable resuscitation, some providers assumed that neonates conceived by in vitro fertilization were “precious babies,” while still others felt that those parents were expecting “perfect babies.”^70^ Such assumptions may influence a provider’s approach to counseling, information sharing, and treatment recommendations.

### Autonomous Decision-Making When There Is Disagreement Between Parents

When there is time to discuss plans for treatment of a periviable infant, involvement of both parents, if there are two parents, is ideal. In a study of periviable decision-making, the majority of clinicians surveyed agreed that partners should be included in the decision-making process. However, when asked who should have decisional authority when there is disagreement between the pregnant person and their partner, nearly all placed “more weight on the pregnant person’s preference.”^71^ Clinicians were often “torn” when asked who should have ultimate decision-making authority in non-traditional families. They were split on whether the pregnant person or the adoptive parents should have the authority to make periviable treatment decisions.^72^ This split demonstrates that clinicians may struggle with how to navigate the balance between respecting the pregnant person’s autonomous decision-making authority as the patient with affording intended parents decision-making rights prior to birth. As noted earlier, since most state laws only confer legal decision-making rights to adoptive parents after birth, the pregnant person would legally have the exclusive authority to make decisions until birth. Notably, clinicians in the study were also more hesitant to assign autonomous decision-making authority to the pregnant person when that person was a surrogate or did not have a biological connection to the fetus.^73^

When consulting parents about decision-making authority, both expectant parents and those who have previously experienced a periviable birth consistently acknowledge the pregnant person as central to the decision-making process, even during conflicts, underscoring their autonomy and recognizing that they bear primary responsibility for the outcomes of the pregnancy. Moreover, when asked to consider situations of adoption and surrogacy, parents overwhelmingly prioritize the individual(s) expected to care for the child in the long term.

## Legal and Ethical Issues in Periviable Treatment Decisions in the Current Climate

### Best Interests v. Vitalism

Laws governing decisions about withholding or withdrawal of care for adults facing a poor prognosis support patient autonomy by allowing patients themselves to determine what treatment they want or do not want. In the event they become incapacitated, they may select a proxy — a trusted surrogate decision-maker. Most states require that surrogate decision-makers apply “substituted judgment” — consenting to treatment or withholding or withdrawal of treatment based on what the patient would have chosen. If the patient’s wishes are unknown or difficult to determine, the proxy should apply a best interests of the patient standard.^74^ In the neonatal context, it is impossible for parents, who essentially serve as proxies for their infant, to substitute judgment; they, therefore, must apply a best interests of the infant approach.^75^

Treatment decisions for periviable infants are deeply challenging for both clinicians and parents. Despite policy efforts that seek to constrain clinical judgement or limit parental authority, clinicians and parents face complex and agonizing treatment decisions every day. Vitalist laws — those that mandate life-sustaining treatment regardless of prognosis — frequently imply that choosing palliative care reflects a lack of moral integrity or coercion among providers. Yet research suggests that, in practice, parents frequently opt for intervention, suggesting that parents are free to decline comfort care in the perinatal or neonatal context.^76^

At the same time, disability law plays an important role in ensuring that decisions are free from bias. Statutes like the Americans with Disabilities Act and Section 504 of the Rehabilitation Act aim to prevent discrimination against people with disabilities and to recognize their dignity and self-determination. Prenatal decision-making about resuscitation raises important ethical questions about how to assess quality of life before birth. The clinical community, led by the AAP, continues to support a shared decision-making — grounded in a best interests standard — that allows families and clinicians to evaluate each case individually, balancing likely outcomes, suffering, and the values of those most affected.

Historically, the law has recognized and upheld parental authority in making medical decisions for their children. Yet laws like Arizona’s proposed mandate for universal resuscitation at birth — regardless of prognosis or parental preference — undermine that authority and strip clinicians of the ability to engage in collaborative decision-making with families.

In early 2025, President Trump endorsed the *Born-Alive Abortion Survivors Protection Act.* ^77^ While not directly focused on periviable care, the bill reflects how the politics of abortion impact discussions of perinatal treatment. In the post-*Dobbs* era, federal and state lawmakers appear more willing to regulate perinatal medicine — often at the expense of clinical judgment and parental autonomy. This shift is especially concerning as some states move to assign fetuses independent legal rights, using both abortion-related and unrelated laws to justify intervention.^78^ Such developments risk undermining the role of parents — those most intimately affected and best positioned to advocate for their child’s well-being — in decisions about life-sustaining treatment. In some cases, that includes the painful but compassionate choice not to prolong an infant’s suffering.

### Decision-Making When Parents Disagree

State laws offer little guidance on how to resolve disagreements between parents about periviable treatment decisions. While some courts have supported parents who choose not to sign a DNR for a newborn, the urgency of resuscitation decisions often makes court involvement impractical. In heterosexual married couples, clinicians tend to view the pregnant patient as having ultimate decision-making authority, while at the same time believing that having both partners engaged is important.^79^

More complex legal and ethical challenges arise when the pregnant person is not the intended parent (i.e., surrogacy and adoption). The law often defaults to biological parentage, while also allowing for intention in parentage determinations, though typically only after birth. This creates a grey area in periviable care, where critical decisions may be needed prenatally before adoptive parents or intended parents’ legal rights are established. While laws that protect surrogate and birth parents’ legal rights until some period after birth are intended to protect bodily autonomy, they do not account for the nuanced question of who should decide whether to resuscitate a periviable infant: the person giving birth or the parent(s) who will ultimately care for the child.

LGBTQ parents without a biological tie to the fetus may also be excluded from these decisions if they lack legal recognition before birth. This exclusion flies in the face of best practices, which promote engagement of both parents in shared decision-making. Studies suggest that, like situations involving surrogacy or adoption, clinicians and parents themselves are torn about which parents to prioritize for decision-making authority — the pregnant person, those with biological ties to the infant or intended parents.^80^

### Considerations for Clinicians

The medical community has supported shared decision-making as the best practice for engaging patients and their families in care decisions. Certainly, in cases involving the unique challenges of deciding whether or not to resuscitate a periviable infant, clinicians should do their best to engage patients and their partners by ensuring that all parents — biological, intended and involved — are informed of prognosis and treatment options, including palliative care, and that all parties are respected and supported.^81^

If the pregnant person and their partner or the intended parents disagree about a resuscitation decision, clinicians and/or hospital staff should attempt to mediate the dispute, but if a resolution cannot be reached, engagement of alternative decision-makers, such as the hospital ethics committees, may be required. Ideally, hospital ethics committees can help to “clarify disputes, hear all viewpoints and discuss options,” helping to move toward resolution.^82^ Courts are a last resort for cases that reach an impasse. In the antenatal context in which a pregnant person is in labor, time may make engagement of either an ethics committee or court unrealistic. In this case, court precedent suggests that resuscitation would be favored.

### Considerations for Lawmakers

State laws, such as the one proposed in Arizona, that restrict parental decision-making about resuscitation of a periviable infant have been framed as protecting the infant from indifferent parents. But legislators do not witness the agony that parents can experience in making these decisions, nor the suffering that some neonates may endure in the resuscitation attempt, only to ultimately not survive the attempt. They fail to recognize that palliation can be a compassionate and loving choice for a parent to make for their child, affording them a peaceful, painless death.

Instead of mandating resuscitation in all cases, lawmakers should work with clinical experts to develop reasonable guidelines that support well-informed, individualized decision-making. Empowering parents, particularly in collaboration with their clinicians, to make choices based on the best interest of the infant allows for nuance and compassion. Although some legislators may fear that this approach leaves too much discretion to parents and clinicians, laws that impose a one-size-fits-all mandate ignore the complexity of real-world medical care. To better support shared decision-making and promote engagement of non-traditional parents, state legislators should adopt the UPA, which offers pathways for recognizing non-biological parents based on intent and involvement.

Yet given today’s polarized legal and political climate, legislative progress on these fronts remains unlikely. Efforts to expand parental discretion and recognize diverse families face considerable headwinds, particularly as some federal and state leaders pursue policies that restrict LGBTQ rights and narrowly define legal parenthood.

## Conclusion

Parental treatment decisions for periviable infants, especially decisions to withhold treatment at birth, raise a whole host of thorny ethical and legal issues. Generally, federal and many state lawmakers have taken a vitalist approach to regulating these decisions, often threatening clinicians and hospitals with civil and criminal liability if they violate the law. In a post-*Dobbs* world in which legislatures and courts are interjecting their own interpretations of “reasonable medical judgment” to preserve fetal life at all costs, clinical and parental decision-making around periviable delivery management are at risk of becoming more restricted in some states. Advocates should continue to educate policymakers about the complexity of medical decisions for periviable infants and encourage them to promote laws that facilitate shared decision-making between clinicians and parents based on the best evidence available, and that centers patients and families of all types.

## References

[1] The American College of Obstetricians and Gynecologists, “Obstetric Care Consensus No. 6,” Obstetrics & Gynecology 130, no. 4 (2017): 187–199, 10.1097/aog.0000000000002352. 28937572

[2] Evangelia Gkiougki et al. “Periviable Birth: A Review of Ethical Considerations,” Hippokratia 25, no. 1 (2021): 1–7. 35221649 PMC8877922

[3] Ravi Mangal Patel et al., “Survival of Infants Born at Periviable Gestational Ages,” Clinics in Perinatology 44, no. 2 (2017): 287–303, 10.1016/j.clp.2017.01.009. 28477661 PMC5424630

[4] The American College of Obstetricians and Gynecologists, “Obstetric Care Consensus No. 6,” Obstetrics & Gynecology 130, no. 4 (2017): 187–199, 10.1097/aog.0000000000002352. 28937572

[5] Evangelia Gkiougki et al. “Periviable Birth: A Review of Ethical Considerations,” Hippokratia 25, no. 1 (2021): 1–7. 35221649 PMC8877922

[6] Jennifer Blumenthal-Barby et al., “Potential Unintended Consequences of Recent Shared Decision Making Policy Initiatives,” Health Affairs 38, no. 11 (2019): 1876–1881, 10.1377/hlthaff.2019.00243. 31682503 PMC7087349

[7] Ian Vandewalker, “Abortion and Informed Consent: How Biased Counseling Laws Mandate Violations of Medical Ethics,” Michigan Journal of Gender & Law 19, no. 1 (2012): 6–70, https://repository.law.umich.edu/mjgl/vol19/iss1/1/.

[8] Meyer v. Nebraska, 262 U.S. 390 (1923).

[9] Pierce v. Society of Sisters, 268 U.S. 510 (1925).

[10] Troxel v. Granville, 530 U.S. 57 (2000).

[11] See Wallis v. Spencer, 202 F.3d 1126, 1141 (9th Cir. 2000); see also Jensen ex rel. Jensen v. Cunningham, 250 P.3d 465, 484 (Utah 2011); Kanuszewski v. Mich. Dep’t of Health & Hum. Services, 927 F.3d 396, 418 (6th Cir. 2019).

[12] Parham v. J.R., 442 U.S. 584 (1979). 12038354

[13] US v. Skrmetti, 605 US (2025); L.W. v. Skrmetti, 679 F. Supp. 3d 668 (M.D. Tenn. 2023).

[14] Raymond S. Duff and A.G.M. Campbell, “Moral and Ethical Dilemmas in the Special-Care Nursery,” New England Journal of Medicine 289, no. 17 (1973): 890–94, 10.1056/nejm197310252891705. 4729120

[15] In re Infant Doe, No. GU8204-004A (Monroe County Cir., Ind. Apr. 12, 1982).

[16] Discriminating Against the Handicapped by Withholding Treatment or Nourishment; Notice of Health Care Providers, 47 Fed. Reg. 26,027-02 (June 16, 1982). See also, George J. Annas, “The case of Baby Jane Doe: Child Abuse or Unlawful Federal Intervention?,” American Journal of Public Health 74, no. 7, (1984):727–729, 10.2105/ajph.74.7.727. 6742262 PMC1651662

[17] Discriminating Against the Handicapped by Withholding Treatment or Nourishment; Notice of Health Care Providers, 47 Fed. Reg. 26,027-02 (June 16, 1982). 11655888

[18] Bowen v. American Hosp. Ass’n, 476 U.S. 610 (1986).

[19] Bowen v. American Hosp. Ass’n, 476 U.S. 610 (1986), at 611.

[20] Amendments to Child Abuse Prevention and Treatment Act. Public L. No. 98-457, 98 Stat. 1749 (amended 1984). 11686171

[21] Procedures relating to health care for handicapped infants, 45 CFR § 84.55 (1985). 10317829

[22] Craig A. Conway, “Baby Doe and Beyond: Examining Practical Philosophical Influences Impacting Medical Decision-Making on Behalf of Marginally-Viable Newborns,” Georgia State University Law Review. 25, no. 4 (2009): 1097–1175, https://readingroom.law.gsu.edu/gsulr/vol25/iss4/4.

[23] Conway, “Baby Doe and Beyond,” at 1147.

[24] Conway, “Baby Doe and Beyond,” at 1147.

[25] Frank X. Placencia and Laurence B. McCullough, “The History of Ethical Decision Making in Neonatal Intensive Care,” Journal of Intensive Care Medicine 26, no. 6 (2011): 368–384, at 370 21606057 10.1177/0885066610393315

[26] Miller v. HCA, 47 Tex. Sup. J. 12, 118 S.W.3d 758 (2003).

[27] George J. Annas, “Extremely Preterm Birth and Parental Authority to Refuse Treatment — The Case of Sidney Miller,” New England Journal of Medicine 351, no. 20 (2004): 2018–2023 10.1056/NEJMlim041201 15537910

[28] Annas, “Extremely Preterm Birth and Parental Authority to Refuse Treatment.” 10.1056/NEJMlim041201 15537910

[29] Annas, “Extremely Preterm Birth and Parental Authority to Refuse Treatment,” at 2121. 10.1056/NEJMlim041201 15537910

[30] Miller v. HCA, 47 Tex. Sup. J. 12, 118 S.W.3d 758 (2003).

[31] Born-Alive Infants Protection Act of 2021, H.R. Rep. No. 107-186, at 3 (2001).

[32] American Academy of Pediatrics Neonatal Resuscitation Program Steering Committee, “Born-Alive Infants Protection Act of 2001, Public Law No. 107- 207,” Pediatrics 111, no. 3 (2003) at 680–81. 10.1542/peds.111.3.680-a 12612257

[33] Emergency Medical Treatment and Labor Act, 42 U.S.C. § 1395dd.

[34] Memo from Department of Health & Human Services, Centers for Medicare and Medicaid Services, Center for Clinical Standards & Quality/Quality Safety & Oversight Group to State Survey Agency Directors “Interaction of the Emergency Medical Treatment and Labor Act (EMTALA) and the Born- Alive Infants Protection Act of 2002” (April 22, 2005), https://www.cms.gov/medicare/provider-enrollment-and-certification/surveycertificationgeninfo/downloads/qso-05-26.pdf.

[35] Executive Order 13952, Protecting Vulnerable Newborn and Infant Children, 85 Fed. Reg. 62187 (Sep. 25, 2020), https://www.federalregister.gov/documents/2020/10/02/2020-21960/protecting-vulnerable-newborn-and-infant-children

[36] Jennifer C. Kett et al., “A 2020 Executive Order That Threatens Progress in Shared Decision-Making,” Pediatrics 147, no. 5 (2021): e2020038794, 10.1542/peds.2020-038794. 33811178

[37] John Stirling, “Understanding Medical Neglect: When Needed Care is Delayed or Refused,” Journal of Child & Adolescent Trauma 13, no. 3 (2019): 271–276, 10.1007/s40653-019-00260-6. 33088383 PMC7561645

[38] Lois A. Weithorn, “When Does a Minor’s Legal Competence to Make Health Care Decisions Matter?,” Pediatrics 146, no. 1 (2020): 25–32, 10.1542/peds.2020-0818G. 32737229

[39] Juvenile and Domestic Relations Courts, NJ Rev. Stat. § 9:6-8.21(1)(a) (2024).

[40] Children’s Code, Wis. Stat. § 48.02(13) (2025).

[41] Arkansas Juvenile Code, Ark. Code Ann. § 9–27–303 (41)(2024).

[42] Meg Nemeth Ledebuhr, “Parentage and the Modern Family: The Only Constant is Change,” American Bar Association, April 1, 2018, archived at: https://web.archive.org/web/20240714134840/https://www.americanbar.org/groups/family_law/publications/family-advocate/2018/spring/4spring2018-ledebuhr/.

[43] American Bar Association, James D. Pedersen, “The New Uniform Parentage Act of 2017,” American Bar Association, April 1, 2018, https://www.americanbar.org/groups/family_law/publications/family-advocate/2018/spring/4spring2018-pedersen/

[44] See e.g., Rhode Island Uniform Parentage Act, Art. 7: Parentage by Assisted Reproduction, 15 R.I. Gen. Laws § 15-8.1-708 (2020).

[45] 15 R.I. Gen. Laws § 15-8.1-708 (2020)

[46] The UPA does not specify the amount of time required. This decision is left to state courts. 15 R.I. Gen. Laws Art. 7 § 15-8.1-708 (2020).

[47] The UPA does not specify the amount of time required. This decision is left to state courts. 15 R.I. Gen. Laws Art. 7 § 15-8.1-708 (2020).

[48] Ten states have enacted the 2017 version of the UPA: California, Colorado, Connecticut, Hawaii, Maine, Massachusetts, Oregon, Rhode Island, Vermont, and Washington. Four states have introduced bills to enact the UPA. “Parentage Act,” Uniform Law Commission, https://www.uniformlaws.org/committees/community-home?CommunityKey=c4f37d2d-4d20-4be0-8256-22dd73af068f#LegBillTrackingAnchor (as of August 2025).

[49] SC Code 1976 § 44-26-60 (A)(1)-(5) (2024).

[50] See e.g., DC Code § 16-4901 (1993). See also, Medical and mental health care consent, 11 Pa. Stat and Cons. Stat. § 2513 (2000), which states that a parent, guardian, or custodian of a minor may confer upon any adult person who is a relative or family friend the power to consent to medical treatment for the minor.

[51] Heather Skirton and Owen Barr, “Antenatal Screening and Informed Choice: A Cross-Sectional Survey of Parents and Professionals,” Midwifery 26, no. 6 (2010): 596–602, 10.1016/j.midw.2009.01.002. 19250723

[52] See e.g., In re Baby K, 832 F. Supp. 1022 (E.D. Va. 1993).

[53] Discussion of state laws governing decisions related to termination of pregnancy based on prenatal testing demonstrating fetal anomalies is beyond the scope of this paper.

[54] Carter Sherman, “The abortion myths Republicans are recycling to reframe a losing issue,” *Guardian*, September 27, 2023, https://www.theguardian.com/world/2023/sep/27/abortion-myths-republicans.

[55] S.B. 1600, 56^th^ Leg., 1^st^ Reg. Sess. (Ariz. 2023). Available at: https://legiscan.com/AZ/text/SB1600/2023

[56] Basil Varkey, “Principles of Clinical Ethics and Their Application to Practice,” Medical Principles and Practice 30, no. 1 (2021): 17–28, 10.1159/000509119. 32498071 PMC7923912

[57] Frank X. Placencia and Laurence B. McCullough, “The History of Ethical Decision Making in Neonatal Intensive Care,” Journal of Intensive Care Medicine 26, no. 6 (2011): 368–384, 10.1177/0885066610393315. 21606057

[58] Placentia and McCullough, “The History of Ethical Decision Making in Neonatal Intensive Care.” 10.1177/0885066610393315 21606057

[59] Steven R. Leuthner, “Decisions Regarding Resuscitation of the Extremely Premature Infant and Models of Best Interest,” Journal of Perinatology 21, (2001): 193–197, 10.1038/sj.jp.7200523 11503107

[60] Daniel S. Goldberg, “The Ethics of DNR Orders as to Neonatal & Pediatric Patients: The Ethical Dimension of Communication,” Houston Journal of Health Law & Policy 7 (2006): 57–83, https://ssrn.com/abstract=936986

[61] Conway, “Baby Doe and Beyond,” at 1120.

[62] Goldberg, “The Ethics of DNR Orders as to Neonatal and Pediatric Patients.”

[63] Brownsyne Tucker Edmonds et al., “Prospective Parents’ Perspectives on Antenatal Decision Making for the Anticipated Birth of a Periviable Infant,” Journal of Maternal-Fetal & Neonatal Medicine 32, no. 5 (2017): 820–5, 10.1080/14767058.2017.1393066. 29103318 PMC6810652

[64] Anuli Njoku et al.,“Listen to the Whispers before They Become Screams: Addressing Black Maternal Morbidity and Mortality in the United States,” Healthcare 11, no. 3 (2023): 1–17, 10.3390/healthcare11030438. PMC9914526 36767014

[65] Yousra A. Mohamoud et al., “Vital Signs: Maternity Care Experiences — United States, April 2023,” Centers for Disease Control and Prevention Morbidity and Mortality Weekly Report 72, no. 35 (2023): 961–967, 10.15585/mmwr.mm7235e1. 37651304

[66] Stephanie Glover et al., Listening to Black Mothers in California (National Partnership for Women and Families, 2018), report available at: https://nationalpartnership.org/health-justice/maternal-health/listening-to-mothers-ca/.

[67] Latoya Hill, et al., “Racial Disparities in Maternal and Infant Health: Current Status and Efforts to Address Them,” Kaiser Family Foundation, October 25, 2024, https://www.kff.org/racial-equity-and-health-policy/racial-disparities-in-maternal-and-infant-health-current-status-and-efforts-to-address-them/.

[68] Dorothy E. Roberts, Torn Apart: How the Child Welfare System Destroys Black Families--and How Abolition Can Build a Safer World (Basic Books, 2022).

[69] DeAnna Y. Smith and Alexus Roane, “Child Removal Fears and Black Mothers’ Medical Decision-Making,” Sage Journals 22, no. 1. (2023):18–23, 10.1177/15365042221142834.

[70] Brownsyne Tucker Edmonds et al., “Predictors of Cesarean Delivery for Periviable Neonates,” Obstetrics & Gynecology 118, no. 1, (2011): 49–56, 10.1097/AOG.0b013e31821c4071. 21691162

[71] Erika R. Cheng et al., “Reflections on Ethics and Advocacy in Child Health Periviable Decision-Making in a New Era of Parentage: Ethical and Legal Considerations and Provider Perspectives on Shared Decision-making in Diverse Family Structures,” Journal of Pediatrics 251 (2022): 24–29, 10.1016/j.jpeds.2022.08.002. 35948190

[72] Cheng, et al., “Reflections on Ethics and Advocacy in Child Health Periviable Decision-Making in a New Era of Parentage,” at 27.

[73] Cheng, et al., “Reflections on Ethics and Advocacy in Child Health Periviable Decision-Making in a New Era of Parentage,” at 27.

[74] Health Care Decision-Making Authority: What is the Decision-Making Standard, (ABA Commission on Law and Aging, July 2015), https://www.americanbar.org/content/dam/aba/administrative/law_aging/What_is_the_Decision_Making_Standard.pdf

[75] Goldberg, “The Ethics of DNR Orders as to Neonatal and Pediatric Patients,” at 69-70.

[76] Goldberg, “The Ethics of DNR Orders as to Neonatal and Pediatric Patients,” at 66.

[77] Executive Office of the President Office of Management and Budget, “Statement of Administration Policy H.R. 21 – Born-Alive Abortion Survivors Protection Act,” White House, January 23,. 2025.

[78] Johanna Hussain, “The Legal Consequences of the Fetal Personhood Movement,” *The Issue Spotter — JLPP Blog*, *Cornell Journal of Law and Public Policy*, (Mar. 4, 2025), https://jlpp.org/legal-consequences-of-the-fetal-personhood-movement/.

[79] Cheng et al., “Reflections on Ethics and Advocacy in Child Health Periviable Decision-Making in a New Era of Parentage:”: 24-29.

[80] Cheng et al., “Reflections on Ethics and Advocacy in Child Health Periviable Decision-Making in a New Era of Parentage:”: 24-29.

[81] Conway, “Baby Doe and Beyond,” at at 1168-1169.

[82] Alex C. Vidaeff and Joseph W. Kaempf, “The Ethics and Practice of Periviability Care,” Children 11, no. 4 (2024): 386–393, 10.3390/children11040386. 38671603 PMC11049503

